# Synthesis of Linear Modified Siloxane-Based Thickeners and Study of Their Phase Behavior and Thickening Mechanism in Supercritical Carbon Dioxide

**DOI:** 10.3390/polym17192640

**Published:** 2025-09-30

**Authors:** Pengfei Chen, Ying Xiong, Daijun Du, Rui Jiang, Jintao Li

**Affiliations:** 1 Research Institute of Natural Gas Technology, PetroChina Southwest Oil & Gas Field Company, Chengdu 610500, China; chenpengfei@petrochina.com.cn (P.C.); xiong_y@petrochina.com.cn (Y.X.); jr950507@163.com (R.J.); 2 Shale Gas Evaluation and Exploitation Key Laboratory of Sichuan Province, Chengdu 610213, China; 3 State Key Laboratory of Oil and Gas Reservoir Geology and Exploitation, Southwest Petroleum University, Chengdu 610500, China

**Keywords:** supercritical CO_2_ fracturing fluid, phase behavior, thickening property, molecular simulation, thickening mechanism

## Abstract

To address critical limitations of ultra-low viscosity supercritical CO_2_ fracturing fluids, including excessive fluid loss and inadequate proppant transport capacity, a series of thickeners designed to significantly enhance CO_2_ viscosity were synthesized. Initially, FT-IR and ^1^H NMR characterization confirmed successful chemical reactions and incorporation of both solvation-enhancing and -thickening functional groups. Subsequently, dissolution and thickening performance were evaluated using a custom-designed high-pressure vessel featuring visual observation capability, in-line viscosity monitoring, and high-temperature operation. All thickener systems exhibited excellent solubility, with 5 wt% loading elevating CO_2_ viscosity to 3.68 mPa·s. Ultimately, molecular simulations performed in Materials Studio elucidated the mechanistic basis, electrostatic potential (ESP) mapping, cohesive energy density analysis, intermolecular interaction energy, and radial distribution function comparisons. These computational approaches revealed dissolution and thickening mechanisms of polymeric thickeners in CO_2_.

## 1. Introduction

With the rapid development of the modern industrial society worldwide, the use of fossil fuels such as crude oil and natural gas has gradually increased, resulting in a large amount of CO_2_ emissions [1]. In response to the rapid growth of CO_2_ emissions, more attention has been paid to CO_2_ capture, utilization, and storage (CCUS) research [2,3]. The global CCUS technologies have been developing for more than 40 years and has been slow to develop due to high investment costs and policy influences [4]. In recent years, there have been developments that can re-create value after CO_2_ capture and storage, such as using CO_2_ to flood oil [5].

With the advancement of oil and gas field development technology, the development of unconventional oil and gas resources is receiving increasing attention [6]. Compared with the problems of reservoir permeability damage exposed by traditional hydraulic fracturing technology, supercritical CO_2_ fracturing technology, which has the advantages of avoiding clay swelling and water locking, enhancing fracturing expansion, easy backflow, and reducing water consumption, is regarded as an ideal fracturing technology with great application potential [7,8]. However, the widespread application of supercritical CO_2_ fracturing is severely limited due to issues such as gravity separation, viscous fingering, and poor sand-carrying capacity caused by its extremely low viscosity during the fracturing process [9].

To solve the above series of problems caused by supercritical CO_2_ fracturing, many scholars have conducted research on CO_2_ thickening; thickening technology refers to the process of dissolving thickening agent such as fluoropolymer, siloxane polymer, hydrocarbon polymer, and surfactant dissolved in CO_2_, and forming a transparent and stable homogeneous phase, so as to achieve the purpose of thickening CO_2_ [6,10,11]. Among them, the solubility and thickening ability of thickeners are of paramount importance in the study of thickeners [9]. Researchers typically seek structural balance between CO_2_-philic and CO_2_-phobic group to achieve a dynamic balance of solubility and thickening ability [10]. Because of its strong interaction with CO_2_ and good solubility, fluoropolymers have become the main CO_2_ solubilizing structure and CO_2_-philic substance [12,13]. Dai et al. [4] prepared a copolymer of heptafluorodecyl acrylate and styrene [P(HFDA/STY)] as a thickener for supercritical CO_2_. The unique binding mode between phenyl groups on the copolymer chains and CO_2_, as well as the molecular simulation radial distribution function (RDF), indicate that phenyl groups have higher aggregation and thickening abilities compared to fluorinated groups. Kilic et al. [14] studied the influence of the composition of copolymer of aromatic acrylate–fluoroacrylate on the viscosity of CO_2_. It was found that the copolymer composed of 29% aromatic acrylate and 71% fluoroacrylate was the most effective CO_2_ thickener. At a pressure of 15 MPa and temperature of 295 K, the thickener is miscible with CO_2_, and the viscosity increased to a certain extent. While achieving a balance between the thickening and solubilizing structure of polymer thickeners, less attention has been paid to the mechanisms of thickening and solubilizing polymer and the interaction between various functional groups and CO_2_.

On the basis of modified siloxane as a thickening group, this study introduces fluorinated groups as solubilizing groups to synthesize fluorinated modified siloxane thickeners. The solubility and thickening ability of fluorinated groups, modified siloxanes, and their copolymers were tested and characterized by infrared and nuclear magnetic resonance, and the microscopic behavior of fluorinated groups, modified siloxanes, and their copolymers in supercritical CO_2_ was studied using full atomic molecular dynamics simulation. The mechanism of action of different groups was explored, providing ideas for the molecular design of CO_2_ thickeners in the future.

## 2. Chemicals and Methods

### 2.1. Chemicals

Concentrated sulfuric acid, sodium carbonate, trimethylolpropane trimethacrylate, isocyanuric acid triallyl ester, tetramethyldisiloxane (HMM), octamethylcyclotetrasiloxane (D4), azodiisobutyronitrile, and chloroplatinic acid were purchased from Chengdu Kelong Co., Ltd., Chengdu, China. All the chemicals were used as received without further purification. CO_2_ and N_2_ were sourced from Chengdu Xindu Jinnengda Gas Co., Ltd., Chengdu, China.

### 2.2. Synthesis of Linear Modified Silicone-Based Supercritical CO_2_ Thickener

#### 2.2.1. Preparation of Silicon Hydrogen-Terminated Linear Polysiloxane

**Figure 1:**
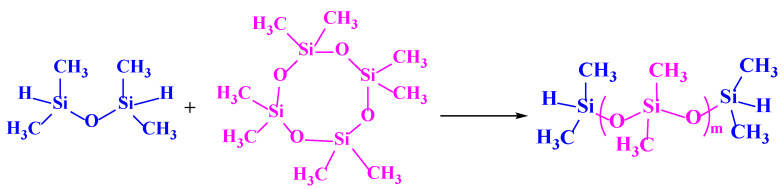
Synthetic routes of HMM-D4.

Under the conditions of 90 °C and concentrated sulfuric acid as a catalyst, 1 g of HMM and 50 g of D4 were polymerized in a N_2_ atmosphere for 12 h to obtain silicon hydrogen-terminated linear siloxane. Subsequently, sodium carbonate was added to filter out the sulfuric acid catalyst, followed by vacuum distillation to remove impurities, resulting in a silicon hydrogen-terminated linear polysiloxane, labeled HMM-D4. The synthesis route is shown in Figure 1.

#### 2.2.2. Synthesis of Supercritical CO_2_ Copolymer Thickeners

**Figure 2:**
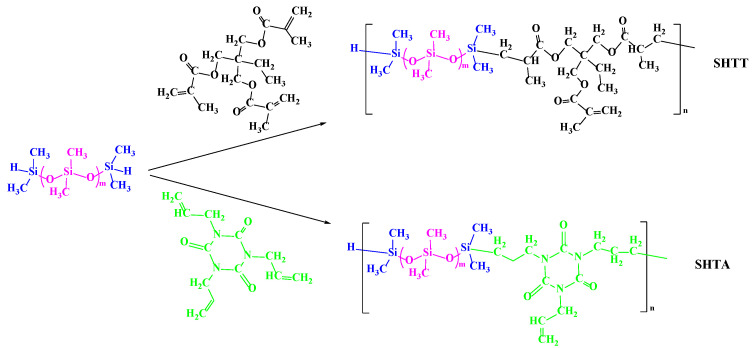
Synthetic routes of copolymer thickeners.

A quantity of trimethylolpropane triacrylate or triallyl isocyanurate was added to a three-necked flask, stirred and heated to 70 °C, and then chloroplatinic acid was added for activation for 2 h. Subsequently, a certain amount of HMM-D4 was added to the flask (the molar ratio of siloxane to acrylic ester was 1:2) under stirring conditions. After 4 h, chloroplatinic acid was separated by precipitation with anhydrous methanol, and the low boiling impurities were removed by vacuum distillation on a rotary evaporator to obtain a yellow liquid, which was the final copolymer thickener, named SHTT or SHTA. The synthesis route is shown in Figure 2.

#### 2.2.3. Modification of SHTT and SHTA

**Figure 3:**
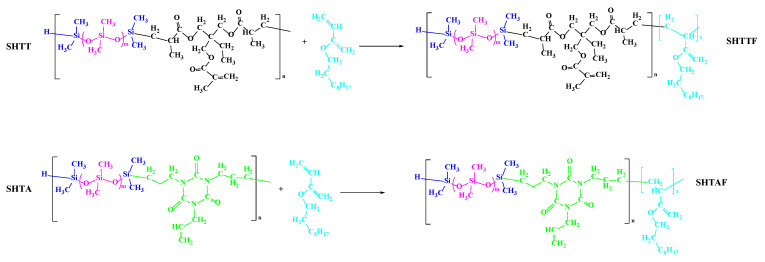
Modification routes of copolymer thickeners.

An amount of 0.001 g of azobisisobutyronitrile was added to 25 g of SHTT or SHTA. After activation for 2 h, 3.885 g of fluorinated acrylate was added under stirring conditions. The modified SHTT and SHTA product was obtained by condensation reflux reaction at a temperature of 80 °C for 6 h and named SHTTF and SHTAF. The modification route is shown in Figure 3.

### 2.3. Characterization

The chemical structure of copolymers and modified copolymers was characterized by FT-IR and ^1^H NMR. The FT-IR spectrum of the copolymers was recorded using a Nicolet Nexus 170SX FT-IR (Madison, WI, USA) on KBr tablets. The ^1^H NMR spectrum of copolymers and modified copolymers were investigated using a Bruker 400 MHZ NMR spectrometer (Japan Electronics Corporation, Tokyo, Japan) using deuterated chloroform as the solvent.

### 2.4. Solubility and Thickening Properties of Copolymers and Modified Copolymers

**Figure 4:**
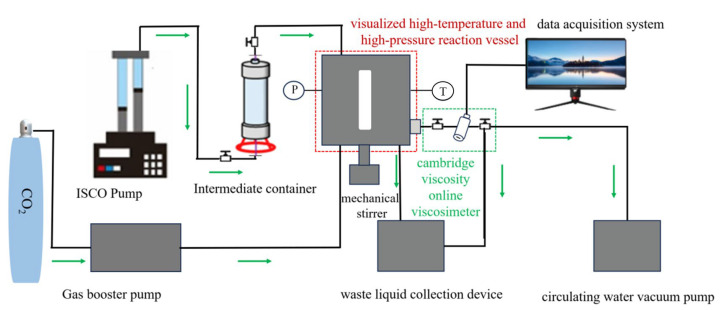
Schematic diagram of visualization supercritical CO_2_ fracturing fluid evaluation device.

The solubility and thickening properties of copolymers and modified copolymers were investigated using a visualization supercritical CO_2_ fracturing fluid evaluation device. The schematic diagram of the experimental device is shown in Figure 4. The experimental steps were as follows.

(1) Accurately weigh the required thickener and add it to the intermediate container.
(2) Seal the visual reaction vessel and heat it to the measurement temperature.
(3) Inject CO_2_ to 0.5 MPa and then reduce the pressure. Repeat this cycle 5 times to expel the air inside the visual reaction vessel.
(4) Use an ISCO pump to inject the weighed thickener in the intermediate container into the visual reaction vessel.
(5) Use a CO_2_ gas booster pump to pressurize CO_2_ to the pressure required for the experiment. The physical property parameters of CO_2_ were referenced from the software REFPROP (version 9.1).
(6) Start mechanical stirring at the bottom of the visual reaction vessel (stirring speed range: 0–1350 rpm). Stir at a constant speed for at least 40 min to promote the dissolution of the thickener and form a transparent and homogeneous solution. (The dissolution of the thickener in supercritical CO_2_ was observed through the front viewing window using an external LED light source placed behind the rear viewing window. High solubility of the thickener was indicated when the mixed fluid inside the visual reaction vessel became clear (the reference object was clearly visible).)
(7) A circulating water vacuum pump was used to evacuate the Cambridge online viscometer.
(8) Open the intake valve of the online viscometer to allow the completely dissolved thickener solution from step (6) to enter. Meanwhile, manually and slowly adjust the volume of the visual reaction vessel to maintain a stable system pressure. Use a data acquisition system to obtain viscosity data.
(9) After the test is completed, direct the system into the waste liquid collection device. Simultaneously, clean the equipment and wait for the next experiment.

### 2.5. Molecular Simulation Study

Molecular simulation techniques were employed to investigate the thickening mechanisms of supercritical CO_2_ thickener, utilizing the Material Studio (version 2023) software package. The Compass force field, which is widely applied in recent years and known for its high accuracy, was selected to calculate the interparticle interactions [5,15,16,17]. Within the Amorphous Cell module, simulation systems containing CO_2_ molecules and modified silicone-based thickener molecules were constructed. Three-dimensional periodic boundary conditions were applied to simulate the supercritical state. The system underwent sequential structural optimization and annealing to obtain the minimum energy conformation. Subsequently, equilibrium molecular dynamics (MD) calculations were performed under the NPT ensemble for both CO_2_ and the thickener polymer, resulting in stable structures for subsequent studies [5,18]. Table 1 lists the detailed information and optimized structures of the different monomers and modified silicone-based thickeners used in the simulations. The simulation process is listed below:

(1) A model was constructed based on the molecular structures presented in Table 1. Force field charges were assigned, and the configuration with the lowest energy was selected for geometric optimization using the Forcite module.
(2) The model obtained from the initial geometric optimization was subjected to annealing using the Forcite module. The system went through 10 annealing cycles, starting from 313.15 K, ramping up to 500 K, and then returning to 313.15 K to reach system equilibrium.
(3) Under the NPT ensemble, the desired temperature and pressure conditions (305.15 K, 10 MPa) were set. The Andersen thermostat and Berendsen barostat were employed, respectively. The time step was set to 1 fs, and the total simulation duration was 500 ps. In the aforementioned molecular dynamics process, one frame was output every 5 ps, and the data from the last 100 ps were used for subsequent data analysis.

#### 2.5.1. Cohesive Energy Density (CED)

It is defined as the energy required to overcome the intermolecular force in 1 mole of a condensed matter substance quantitatively characterized in the interactions between polymer molecules [19]. And the solubility parameter (*δ*) is defined as the square root of CED [20].

(1) $$E=-E_{inter}=E_{van}+E_{elect}+E_{other}$$

(2) $$CED=\frac{E}{V}$$

(3) $$\delta =\sqrt{CED}=\sqrt{\frac{E}{V}}$$

where the CED is the cohesive energy density, J/m^3^; *E* is the energy of the substance, kJ/mol; *V* is the molar volume of the substance, L/mol; *δ* is the solubility parameter, (J/m^3^)^1/2^; *E_inter_* is the total intermolecular energy of the system (kJ/mol); *E_van_* is the energy from van der Waals interactions (kJ/mol); and *E_elect_* is the energy from electrostatic interactions (kJ/mol).

#### 2.5.2. Interaction Energy

The interaction energy serves to quantitatively characterize the strength of interactions between the polymer and CO_2_. A larger absolute magnitude of the interaction energy indicates stronger interactions between the polymer and CO_2_ [1].

(4) $$E_{int}=E_{polymer+{CO}_{2}}-(E_{polymer}+E_{{CO}_{2}})$$

where *E_int_* is the polymer–CO_2_ interaction energy, (kJ/mol); *E_polymer+CO_*_2_ is the total energy of the polymer–CO_2_ system (kJ/mol); *E_polymer_* is the energy of the polymer (kJ/mol); and *E_CO_*_2_ is the energy of CO_2_, kJ/mol.

#### 2.5.3. Radial Distribution Function (RDF)

The RDF is commonly used to investigate the microscopic structure of systems and evaluate intermolecular interactions. It typically represents the probability density of finding a molecule (or atom) of *A* at a specific distance from a molecule (or atom) of *B* [21] calculated as

(5) $$g_{AB}(r)=\frac{1}{\rho_{AB}\cdot 4\pi r\cdot {∆}_{r}}\frac{\sum_{j=1}^{N_{AB}}{∆N}_{AB}(r\to r+∆r)}{N_{AB}}$$

where $g_{AB}(r)$ is the radial distribution function value; $N_{AB}$ is the number of species *A* and *B* (molecules or atoms) in the system; ∆r is the distance interval width; ${∆N}_{AB}$ is the number of *B* particles (or *A* particles) found within the distance range *r*~*r* + Δ*r* from a central *A* particle (or *B* particle); and $\rho_{AB}$ is the density of system.

## 3. Results and Discussion

### 3.1. Characterization of Supercritical CO_2_ Thickener

#### 3.1.1. FT-IR

**Figure 5:**
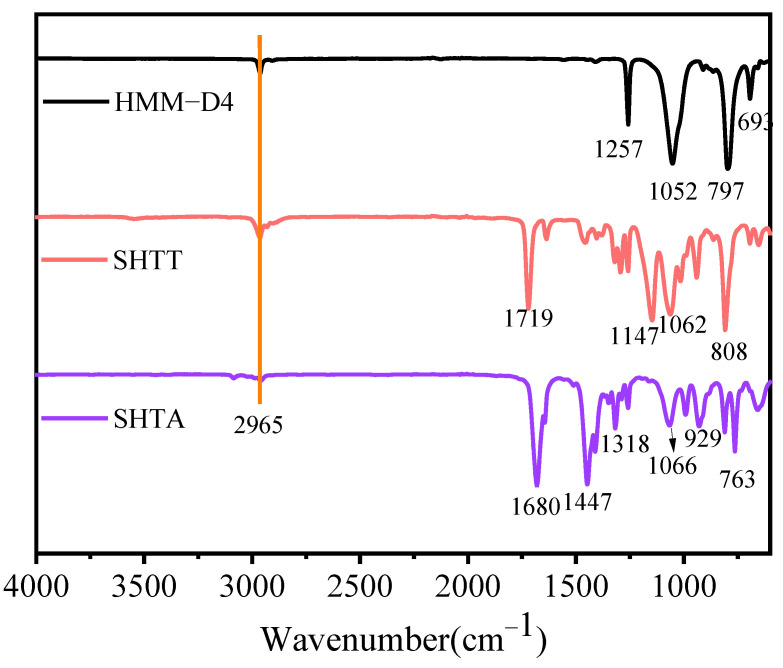
FTIR spectra of HMM-D4, SHTT and SHTA.

Figure 5 shows the FT-IR spectra of HMM-D4, SHTT, and SHTA. The FT-IR spectra of HMM-D4 primarily exhibit characteristic peaks including the Si-CH_3_ stretching vibration at 1257 cm^−1^, Si-O stretching vibration at 1064 cm^−1^ and 1052 cm^−1^, and the Si-C stretching vibration at 797 cm^−1^. As the hydrosilylation reaction progresses, the characteristic peaks of HMM-D4 are still retained in the infrared spectra of the obtained thickeners SHTT and SHTA. However, the intensities of the peaks corresponding to the Si-CH_3_ stretching vibration at 1257 cm^−1^ and the Si-O stretching vibration at 1052 cm^−1^ are significantly reduced [22]. Additionally, new peaks emerge at 1680 cm^−1^ (C=C stretching vibration of CH_2_=CH–), 1719 cm^−1^ (C=O stretching vibration), and 1447 cm^−1^ (bending vibration of saturated C–H bonds in –CH_2_– and –CH_3_ groups) [23,24]. Based on the above analysis, the appearance of new characteristic peaks in the infrared spectra of the thickeners SHTT and SHTA corresponds to the changes in functional groups during the hydrosilylation reaction [17]. This conclusion is consistent with the previous research results of Tang et al. [22] and Liu et al. [17,19].

#### 3.1.2. ^1^H NMR

**Figure 6:**
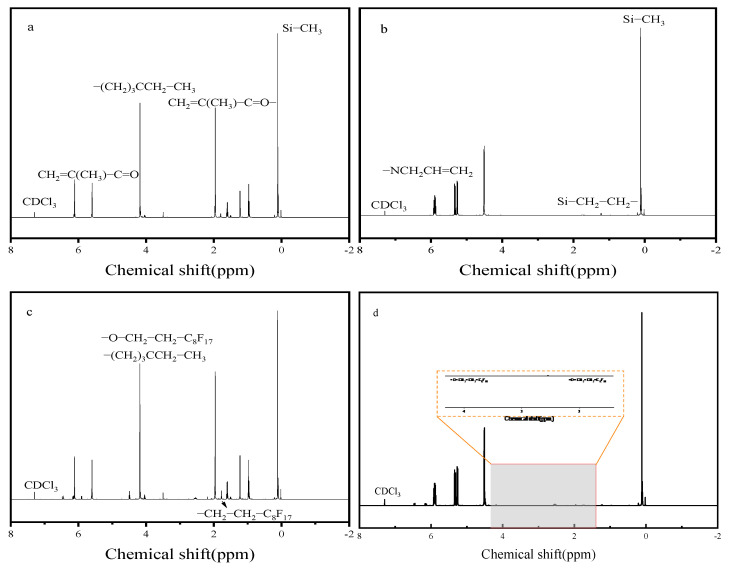
^1^H NMR spectra of SHTT (**a**), SHTA (**b**), SHTTF (**c**), and SHTAF (**d**).

Figure 6 shows the ^1^H NMR spectra of the thickener copolymers. The peak at 7.26 ppm corresponds to the residual protons in the solvent CDCl_3_. For SHTT (Figure 6a), key signals include alkenyl protons (CH_2_=C(CH_3_)C=O–) at 5.5–6.2 ppm, methylene protons (–(CH_2_)_3_CCH_2_CH_3_) at 3.9–4.2 ppm, methyl protons (CH_2_=C(CH_3_)C=O–) at 1.94 ppm, methylene protons of the ethyl group (–(CH_2_)_3_CCH_2_CH_3_) at 1.2 ppm, methylene and methyl protons (O=C–CH(CH_3_)CH_2_Si–) at 0.89–0.98 ppm, and methyl protons bonded to silicon (Si–CH_3_) at 0.1–0.2 ppm. For SHTA (Figure 6b), key signals include methine proton (–NCH_2_CH=CH_2_) at 5.8–5.93 ppm, alkenyl protons (–NCH_2_CH=CH_2_) at 5.2–5.34 ppm, methylene protons of the allyl group (–CH_2_–) at 4.4–4.6 ppm, methylene protons adjacent to silicon (Si–CH_2_CH_2_–) at 1.2–1.6 ppm, and methyl protons bonded to silicon (Si–CH_3_) at 0–0.2 ppm [17,19].

Compared with SHTT (Figure 6a), in SHTTF (Figure 6c), peaks corresponding to the hydrogen atoms of the methylene group adjacent to the oxygen atom in –O–CH_2_–CH_2_–C_8_F_17_ appear at δ = 3.9–4.2 ppm, and peaks corresponding to the hydrogen atoms of the methylene group adjacent to the fluorine atoms in –O–CH_2_–CH_2_–C_8_F_17_ appear at δ = 1.7–1.94 ppm. Similarly, in SHTAF (Figure 6d), the peak corresponding to the hydrogen atoms of the methylene group adjacent to the oxygen atom in –O–CH_2_–CH_2_–C_8_F_17_ appears at δ = 4.2 ppm, and the peak corresponding to the hydrogen atoms of the methylene group adjacent to the fluorine atoms in –O–CH_2_–CH_2_–C_8_F_17_ appears at δ = 1.7–1.96 ppm. Similar to the findings of Dai et al. [4] and Sun et al. [13], the changes in the values of these characteristic peaks correspond to the changes in the corresponding functional groups after the introduction of fluorides. Combining the previous infrared analysis and the above NMR results, it indicates the successful preparation of the thickener copolymers.

### 3.2. Phase Behavior

**Figure 7:**
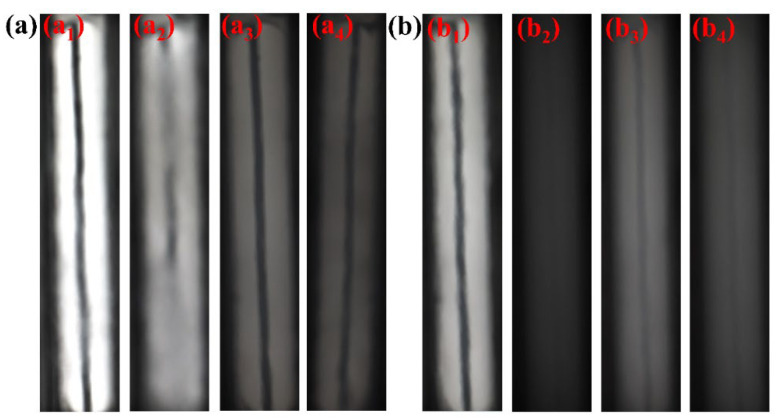
Solubility performance under different temperatures and pressures: (**a**) SHTT-CO_2_ (**a_1_**: 25 °C, 7.5 MPa; **a_2_**: 32 °C, 7.5 MPa; **a_3_**: 32 °C, 10 MPa; **a_4_**: 32 °C, 7.5 MPa); (**b**) SHTTF-CO_2_ (**b_1_**: 25 °C, 7.5 MPa; **b_2_**: 32 °C, 7.5 MPa; **b_3_**: 32 °C, 10 MPa; **b_4_**: 32 °C, 7.5 MPa).

The phase behavior of SHTT and SHTTF in ScCO_2_ was investigated using visualized reaction vessel as shown in Figure 4, with results presented in Figure 7 and Table 2. The observation revealed that the system remained clear and transparent under stirred conditions at 25 °C and 7.5 MPa, allowing distinct visibility of the reference wire behind the observation window (Figure 7(a_1_,b_1_)). When stirring was initiated at 32° C and 7.5 MPa, the system turned turbid as temperature increase intensified molecular thermal motion, promoting gradual dissolution of the thickener and completely obscuring the reference wire (Figure 7(a_2_,b_2_)). Upon maintaining the temperature while slowly increasing pressure to 10 MPa, the thickener became fully dissolved in ScCO_2_, accompanied by brightening of the solubility phase diagram and restored visibility of the reference wire (Figure 7(a_3_,b_3_)). When pressure was reduced back to 7.5 MPa, the system appeared more turbid than in Figure 7(a_3_,b_3_) at 32 °C and 10 MPa but retained better clarity than in Figure 7(a_2_,b_2_), suggesting that although pressure decreased, the polymeric thickener remained dissolved in supercritical CO_2_ without complete precipitation [15]. During this process, the dissolution pressures of SHTT-CO_2_ and SHTTF-CO_2_ were measured to be 9.2 MPa and 8.6 MPa, respectively. The dissolution pressure is defined as the pressure at which the thin-line reference changes from visible to invisible during the pressure reduction process starting from 10 MPa (the measurement is repeated three times, and the average value is taken) [17,19].

### 3.3. Thickening Ability

**Figure 8:**
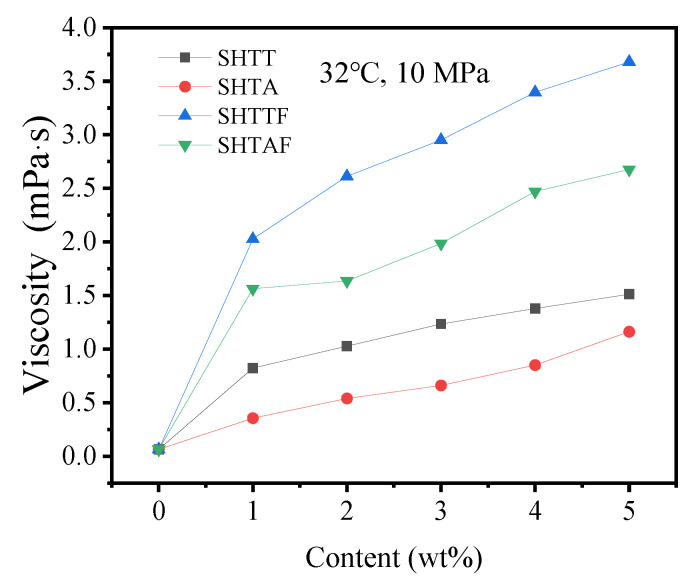
The influence of different contents and types of thickeners on the viscosity of thickened supercritical CO_2_ at 32 °C and 10 MPa.

The thickening ability of SHTT, SHTA, SHTTF, and SHTAF were investigated at 32 °C and 10 MPa, and the results are shown in Figure 8 and Table 3. All ScCO_2_ fracturing fluid systems with different thickeners exhibited the same trend. Based on the literature review, a good thickener should achieve a thickening ratio of 10~100 times (compared to pure CO_2_) at a low concentration (0~5 wt%) and achieve excellent solubility while maintaining thickening performance, cost-effectiveness, and safety [9,10]. Based on this, in this study, the authors will investigate the concentration of the additive (thickener) in the range of 0~5 wt%. The viscosity of ScCO_2_ increased with higher thickener content. However, fluorinated modified copolymers (SHTTF, SHTAF) demonstrated a significantly stronger impact on ScCO_2_ viscosity than SHTT and SHTA, highlighting its superior thickening capacity. For instance, adding 5 wt% SHTTF increased the viscosity of CO_2_ to 3.68 mPa·s. At the initial stage of adding SHTT or SHTA, the system contained only a small number of thickener molecules, resulting in limited van der Waals interactions and hydrogen bonding between CO_2_ molecules and the thickener. This formed a sparse spatial network structure. As the thickener content increased—particularly with fluorinated thickeners like SHTTF and SHTAF—more modified siloxane polymers dissolved in CO_2_. This enhanced the interactions between CO_2_ molecules and thickener molecules, leading to the formation of a denser microscopic spatial network structure [16,19]. Consequently, the apparent viscosity of CO_2_ increased macroscopically.

**Figure 9:**
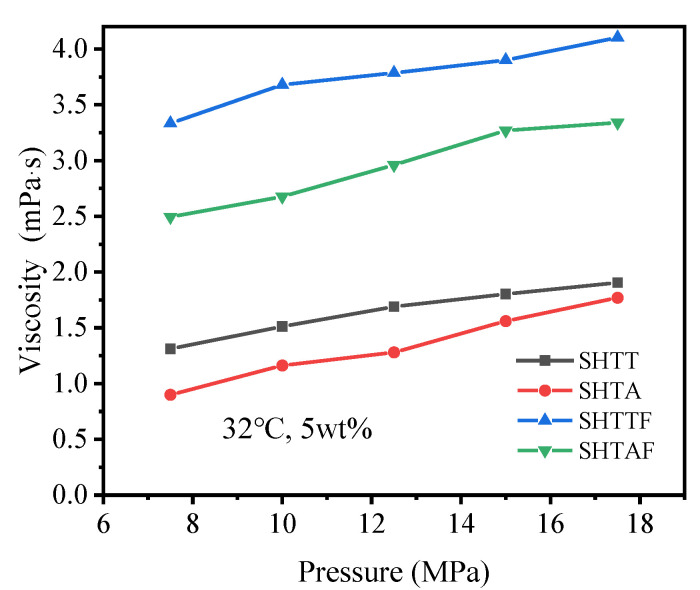
Effect of pressure on thickened CO_2_ viscosity.

Under experimental conditions of 32 °C and 5 wt% thickener dosage, the effect of pressures on the viscosity of CO_2_ thickened by various agents was studied, with results shown in Figure 9 and Table 4. The viscosity of thickened CO_2_ increased with rising pressure, with SHTTF demonstrating the optimal thickening performance. The likely reasons are (1) the incorporation of fluoride groups enhances the solubility of the modified siloxane polymer in CO_2_; (2) during pressurization, decreasing intermolecular distances facilitate the formation of hydrogen bonding through Lewis acid–base pairing. These two effects work synergistically, manifesting macroscopically as an increase in CO_2_ viscosity [19,25].

**Figure 10:**
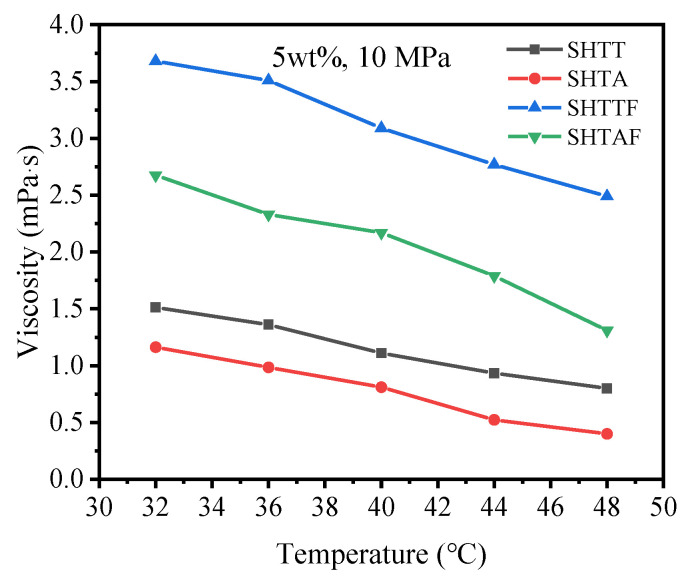
Effect of temperature on thickened CO_2_ viscosity.

Figure 10 presents the curves depicting the influence of temperature on the thickening capacity of various thickeners for the CO_2_ system (Table 5 shows the original data corresponding to Figure 10). The results indicate that the viscosity of CO_2_ thickened by SHTT, SHTA, SHTTF, and SHTAF declined progressively with increasing temperature. This occurs because, at lower temperatures, the three-dimensional network structures formed by molecular intertwining are insufficient to counteract the disruption of the network structure induced by the temperature rise, as well as the detrimental effect on viscosity caused by the breakdown of Lewis acid–base pairs [12,26].

**Figure 11:**
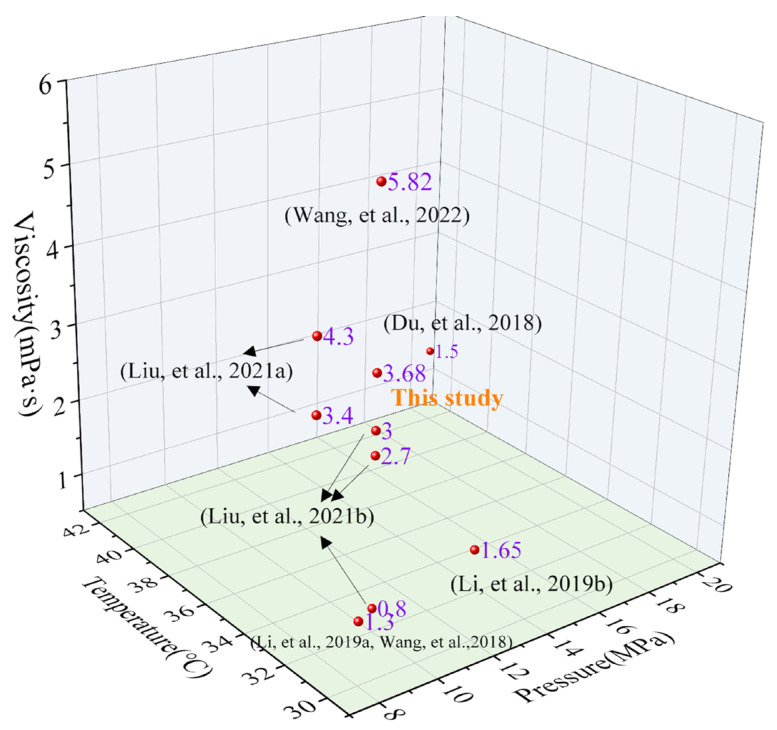
A 3D diagram comparing the viscosity data of this study with those of similar thickeners in the existing literature. The corresponding references in the figure are as follows: Wang, et al., 2022 [16], Liu, et al., 2021a [17], Liu, et al., 2021b [19], Du, et al., 2018 [27], Li, et al., 2019a [28], Wang, et al., 2018 [29], Li, et al., 2019b [30].

To further clarify the gap between the thickener prepared in this study and similar thickeners in the current literature, Table 6 and Figure 11 present the detailed viscosity data of similar thickeners under different experimental conditions in the literature and a 3D intuitive comparison diagram, respectively. Overall, the SHTTF thickener prepared in our study exhibits relatively excellent CO_2_ thickening ability, which is superior to the reported HBD-1 thickener (32 °C/8 MPa) [17], HS series thickeners (32 °C/10 MPa) [19], PDMS (42 °C/20 MPa) [27], EEPDMS (30 °C/8 MPa) [28,29], and Ester-branched polydimethylsiloxane (30 °C/12 MPa) [30]. In addition, it is worth mentioning that, in comparison with the thickeners in these references, no cosolvent was added in this study. However, there is still a significant gap in the thickening ability between the thickener in this study and the reported HBD-2 [17] and SiO_2_/HBD-2 thickeners [16] in the literature, which is exactly the direction of our future efforts (structure optimization, compounding of different types of thickeners, or introduction of nanomaterials).

### 3.4. Thickening Mechanisms

#### 3.4.1. Molecular Electrostatic Potential Map

**Figure 12:**
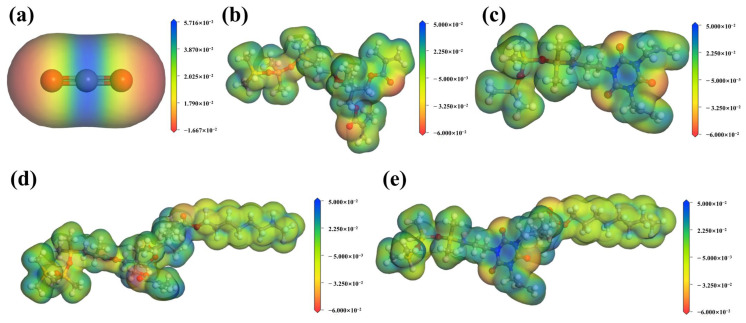
Electrostatic potential distribution of CO_2_ molecules and modified siloxane thickeners ((**a**), CO_2_; (**b**), SHTT; (**c**), SHTA; (**d**), SHTTF; (**e**), SHTAF).

Figure 12 shows the electrostatic potential distribution of CO_2_ molecules and modified siloxane thickeners. In CO_2_, negative charges concentrate on oxygen atoms, whereas positive charges in both modified siloxane and fluorinated modified siloxane thickeners distribute evenly across carbon-containing groups [32]. Simultaneously, the electrostatic potential at polymerization sites between modified siloxane and fluorides (Figure 12d,e) appears slightly higher than that of surrounding functional groups [15]. Consequently, both modified and fluorinated modified siloxane thickeners can bind with CO_2_ molecules through electrostatic attraction [1,15].

#### 3.4.2. CED and Δ

CED and *δ* are commonly employed to characterize intermolecular interactions in polymers. CED is defined as the energy required to vaporize one mole of a condensed substance per unit volume, overcoming intermolecular forces, which primarily reflects inter-group interactions [33]. Relevant studies demonstrate that polymers with lower CED exhibit higher solubility in CO_2_. Furthermore, smaller differences between polymer and CO_2_ solubility parameters correlate with enhanced dissolution performance of the polymer in CO_2_ [34]. The CED and *δ* of CO_2_ and thickened CO_2_ system are tabulated in Table 7, all four thickener polymers show minor *δ* differences with CO_2_. Among these, SHTTF exhibits the smallest δ difference with CO_2_, confirming its optimal solubility in the CO_2_ system.

#### 3.4.3. Intermolecular Interaction Energy

Interaction energy (*E_int_*) quantitatively characterizes the strength of interactions between polymers and CO_2_ molecules (calculated via Equation (4)) [18,35]. A larger absolute value of *E_int_* indicates stronger polymer–CO_2_ interactions, which enhances polymer solubility in CO_2_ [33,36]. As shown in Table 8, in modified siloxane systems, the SHTT-CO_2_ system exhibits a greater absolute *E_int_* than the SHTA-CO_2_ system, confirming superior solubility of thickener polymer SHTT in CO_2_. After fluorination modification, thickener polymer SHTTF maintains stronger solubility in CO_2_ than SHTAF. Based on the above interaction energy data between different thickeners and CO_2_, the solubility order of different thickeners in CO_2_ is as follows: SHTTF-CO_2_ > SHTAF-CO_2_ > SHTT-CO_2_ > SHTA-CO_2_. Among them, the maximum interaction energy between the thickener SHTTF and CO_2_ indicates that it has the strongest polymer–CO_2_ interaction with CO_2_, which improves its solubility in CO_2_ and thickening ability. This result corresponds to the result in the thickening ability test in Section 3.3. In addition, the reason for the enhanced polymer–CO_2_ interaction may be that the interaction between the polar sites on the polymer chain and CO_2_ breaks the intermolecular interaction between polymer–polymer molecules [36].

#### 3.4.4. Radial Distribution Function (RDF)

**Figure 13:**
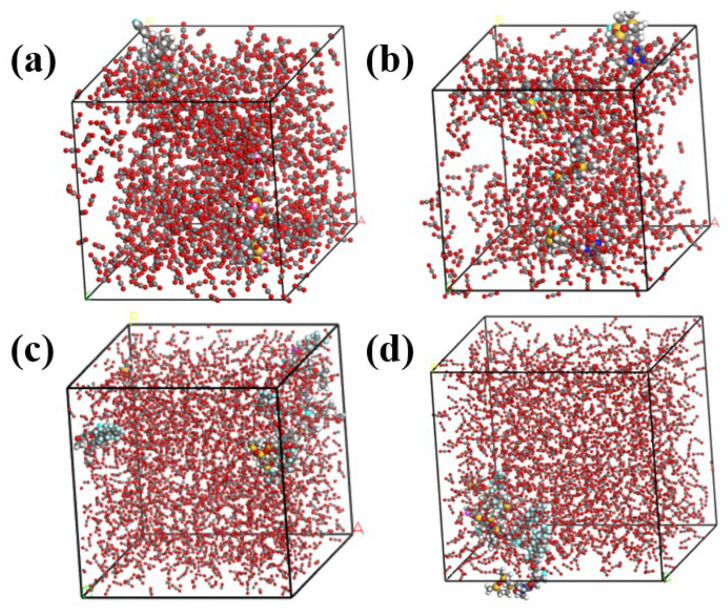
The system model after molecular dynamics optimization ((**a**) SHTT-CO_2_, (**b**) SHTA-CO_2_, (**c**) SHTTF-CO_2_, (**d**) SHTAF-CO_2_)).

**Figure 14:**
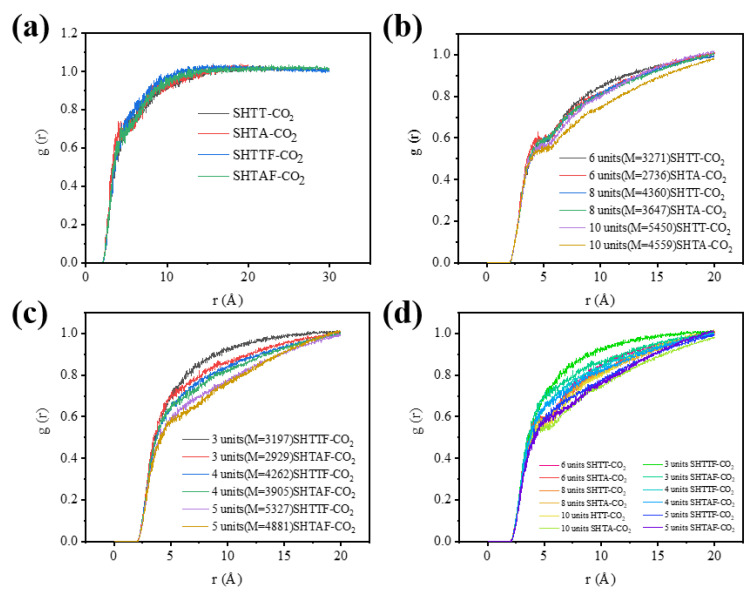
The radial distribution functions of thickeners with different chain lengths and molecular weights and CO_2_ molecules. The compositions of each system are as follows: (**a**) four repeating units of the thickener + 1000 CO_2_ molecules; (**b**) thickener polymer chains consisting of four chains with 6, 8, and 10 repeating units of the thickener (SHTT/SHTA) + 1000 CO_2_ molecules, respectively; (**c**) thickener polymer chains consisting of four chains with 3, 4, and 5 repeating units of the thickener (SHTTF/SHTAF) + 1000 CO_2_ molecules, respectively; (**d**) A combination of the Figure (**b**,**c**).

Calculation of radial distribution functions between different thickener molecules and carbon dioxide molecules by means of trajectory files of molecular dynamics optimized system models (Figure 13a–d). Figure 14 shows the radial distribution functions between different thickeners and CO_2_: (a) four repeating units of the thickener + 1000 CO_2_ molecules; (b) thickener polymer chains consisting of four chains with 6, 8, and 10 repeating units of the thickener (SHTT/SHTA) + 1000 CO_2_ molecules, respectively; (c) thickener polymer chains consisting of four chains with 3, 4, and 5 repeating units of the thickener (SHTTF/SHTAF) + 1000 CO_2_ molecules, respectively; (d) a combination of Figure 13b,c. From the RDFs (Radial Distribution Functions) of different thickeners and CO_2_ in Figure 14, it can be seen that a higher RDF value indicates better miscibility between the thickener and CO_2_. From Figure 14a,d, it can be obtained that the dissolution abilities of thickeners with different types, different chain lengths, and different molecular weights in CO_2_ are in the following order: SHTTF-CO_2_ > SHTAF-CO_2_ > SHTT-CO_2_ > SHTA-CO_2_. That is, the introduction of fluorocarbon chains weakens the intermolecular interactions between the thickener polymer molecules, enhancing their solubility and thickening ability [4,36]. From the RDF data in Figure 14b,c, it can be seen that as the chain length and molecular weight of the thickeners (SHTT/SHTA, SHTTF/SHTAF) increase, their dissolution abilities in CO_2_ decrease. The reason for this phenomenon may be that while the increase in the chain length and molecular weight of the thickener polymer introduces more polar sites, the intermolecular interactions between polymer–polymer molecules also increase, and the interaction between the polymer and CO_2_ weakens, resulting in a decrease in solubility and thickening ability [37].

## 4. Conclusions

(1) Four modified silicone-based thickeners for supercritical CO_2_ fracturing fluids were successfully synthesized. FT-IR and NMR characterization confirmed the successful incorporation of CO_2_-philic groups into thickener molecular structures, achieving the synthesis of fluorinated silicone thickeners.
(2) The fluorinated silicone thickeners demonstrated exceptional thickening performance and favorable solubility. At 5 wt% concentration under 10 MPa and 32 °C, they formed homogeneous mixtures with CO_2_, elevating the viscosity of CO_2_ to 3.68 mPa·s. Systematic experiments established positive correlations between CO_2_ viscosity and both thickener concentration and pressure, while revealing an inverse relationship with temperature. The limitation of this study is that it only investigated the thickener concentrations within the range of 0~5 wt%.
(3) Integrating experimental results with molecular simulations, the thickening mechanism was elucidated: The introduction of fluorinated compounds with carbon dioxide-affinitive moieties significantly enhances the dissolution of thickening functional-modified silicone segments in CO_2_, thereby substantially increasing the viscosity of CO_2_ fracturing fluids.

## Figures and Tables

**Table 1:** The repetitive unit structures of different thickeners (yellow, white, gray, red, blue, and purple denote silicon, hydrogen, carbon, oxygen, fluorine, and nitrogen atoms, respectively).

Name	Molecular Structure
CO_2_	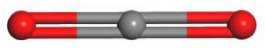
SHTT	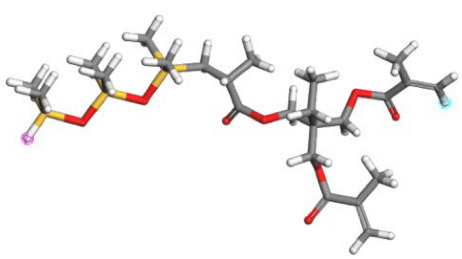
SHTA	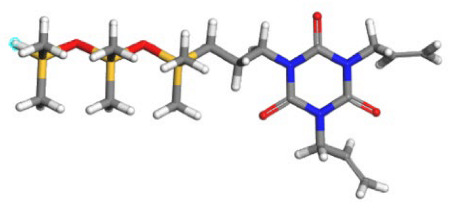
SHTTF	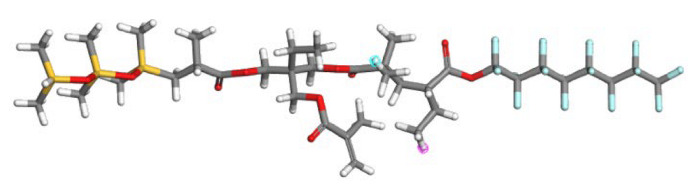
SHTAF	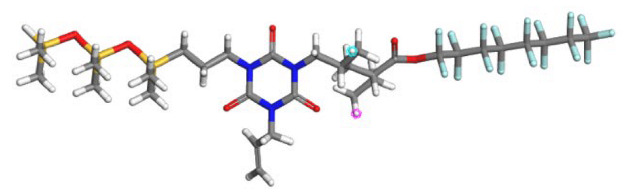

**Table 2:** Phase behavior and dissolution pressure of thickeners (SHTT and SHTTF) in CO_2_ under different temperatures and pressures.

System	Temperature (°C)/Pressure (MPa)				Dissolution Pressure (MPa)
	25 °C, 7.5 MPa	32 °C, 7.5 MPa	32 °C, 10 MPa	32 °C, 7.5 MPa	
SHTT-CO_2_	a_1_	a_2_	a_3_	a_4_	9.2
	Clarified	Turbid, reference object not visible	Clear and transparent	Turbid, reference object visible	
SHTTF-CO_2_	b_1_	b_2_	b_3_	b_4_	8.6
	Clarified	Turbid, reference object not visible	Clear and transparent	Turbid, reference object visible	

**Table 3:** The original viscosity data of supercritical CO_2_ thickened by different contents and types of thickeners at 32 °C and 10 MPa.

Temperature (°C)/Pressure (MPa)		System	SHTT-CO_2_	SHTA-CO_2_	SHTTF-CO_2_	SHTAF-CO_2_
	Content (wt%)		Viscosity (mPa·s)			
32 °C, 10 MPa	0		0.06335 (REFPROP)			
	1		0.823	0.355	2.026	1.564
	2		1.027	0.539	2.612	1.636
	3		1.234	0.66	2.951	1.986
	4		1.378	0.85	3.396	2.47
	5		1.512	1.162	3.68	2.676

**Table 4:** The original viscosity data of supercritical CO_2_ thickened by different types of thickeners under different pressures at 32 °C with a thickener content of 5 wt%.

Temperature (°C)/Content (wt%)		System	SHTT-CO_2_	SHTA-CO_2_	SHTTF-CO_2_	SHTAF-CO_2_
	Pressure (MPa)		Viscosity (mPa·s)			
32 °C, 5 wt%	7.5		1.312	0.9	3.334	2.495
	10		1.512	1.162	3.68	2.676
	12.5		1.69	1.28	3.785	2.96
	15		1.803	1.56	3.9	3.27
	17.5		1.905	1.77	4.102	3.34

**Table 5:** The original viscosity data of supercritical CO_2_ thickened by different types of thickeners at different temperatures under 10 MPa with a thickener content of 5 wt%.

Pressure (MPa)/Content (wt%)		System	SHTT-CO_2_	SHTA-CO_2_	SHTTF-CO_2_	SHTAF-CO_2_
	Temperature (°C)		Viscosity (mPa·s)			
10 MPa, 5 wt%	32		1.512	1.162	3.68	2.676
	36		1.36	0.985	3.51	2.33
	40		1.11	0.81	3.09	2.17
	44		0.934	0.523	2.77	1.79
	48		0.8	0.4	2.49	1.31

**Table 6:** Under different experimental conditions (content, cosolvent, temperature, pressure), the viscosity data of this study are compared with those of similar thickeners in the existing literature [31].

Thickener	Concentration (wt%)	Cosolvent (wt%)	Experimental Condition	Viscosity (mPa·s)	Ref.
HBD-1	5	no	32 °C/8 MPa	3.4	[17]
HBD-2	5	no	32 °C/8 MPa	4.3	
SHTTF	5	no	32 °C/10 MPa	3.68	This study
HS-1	5	no	32 °C/10 MPa	0.8	[19]
HS-2	5	no	32 °C/10 MPa	2.7	
HS-3	5	no	32 °C/10 MPa	3.0	
PDMS (350 mPa·s)	5	10 wt% toluene	42 °C/20 MPa	1.5	[27]
SiO_2_/HBD-2	5 wt% HBD-2 + 1 wt% SiO_2_	no	32 °C/10 MPa	5.82	[16]
EEPDMS	3	9 wt% toluene	30 °C/8 MPa	1.3	[28,29]
Ester-branched polydimethylsiloxane	2.5	7.5 wt% n-hexane	30 °C/12 MPa	1.65	[30]

**Table 7:** CED and *δ* of CO_2_ and thickened CO_2_ at 40 °C and 10 MPa.

System	*E_van_*/(J/m^3^)	*E_elect_*/(J/m^3^)	*E_other_*/(J/m^3^)	CED/(J/m^3^)	*δ*/(J/m^3^)^1/2^	Δ*δ*/(J/m^3^)^1/2^
CO_2_	7.958 × 10^7^	5.629 × 10^7^	3.033 × 10^6^	1.389 × 10^8^	11.777	0
SHTT-CO_2_	1.365 × 10^8^	9.910 × 10^7^	5.454 × 10^6^	2.411 × 10^8^	15.512	3.735
SHTA-CO_2_	1.369 × 10^8^	9.938 × 10^7^	5.413 × 10^6^	2.417 × 10^8^	15.507	3.730
SHTTF-CO_2_	7.202 × 10^7^	4.727 × 10^7^	2.803 × 10^6^	1.221 × 10^8^	11.036	0.741
SHTAF-CO_2_	6.082 × 10^7^	3.986 × 10^7^	2.305 × 10^6^	1.030 × 10^8^	10.119	1.658

**Table 8:** Intermolecular interaction energy between thickener and CO_2_ (4 repeating units of the thickener + 1000 CO_2_ molecules).

System	$E_{{Polymer-CO}_{2}}$/(kcal/mol)	$E_{Polymer}$/(kcal/mol)	$E_{{CO}_{2}}$/(kcal/mol)	*E_int_*/(kcal/mol)
SHTT-CO_2_	−2449.962372	−1406.236231	−846.918705	−196.807436
SHTA-CO_2_	−2205.395479	−1181.816535	−847.726976	−175.851968
SHTTF-CO_2_	−1635.494087	−390.389668	−1028.260001	−216.844418
SHTAF-CO_2_	−1473.056476	−292.508835	−977.561245	−202.986396

## Data Availability

The original contributions presented in this study are included in the article. Further inquiries can be directed to the corresponding author(s).

## Abbreviations

The following abbreviations are used in this manuscript:

CCUS	CO_2_ capture, utilization, and storage
FT-IR	fourier transform infrared spectrum
^1^H NMR	^1^H nuclear magnetic resonance spectroscopy
ESP	electrostatic potential
HMM	tetramethyldisiloxane
D4	octamethylcyclotetrasiloxane
MD	molecular dynamics
CED	cohesive energy density
*E*	the energy of the substance
*V*	the molar volume of the substance
*δ*	the solubility parameter
*E_inter_*	the total intermolecular energy of the system
*E_van_*	the energy from van der Waals interactions
*E_elect_*	the energy from electrostatic interactions
*E_int_*	the polymer–CO_2_ interaction energy
*E_polymer+CO_*_2_	the total energy of the polymer–CO_2_ system
*E_polymer_*	the energy of the polymer
*E_CO_*_2_	the energy of CO_2_, kJ/mol
RDF	radial distribution function
$g_{AB}(r)$	the radial distribution function value
$N_{AB}$	the number of species A and B (molecules or atoms) in the system
∆r	the distance interval width
${∆N}_{AB}$	the number of B particles (or A particles) found within the distance range r~r + Δr from a central A particle (or B particle)
$\rho_{AB}$	the density of system
